# Construction Notes

**Published:** 1905-07-29

**Authors:** 


### CONSTRUCTION NOTES.

The Committee of the "Dumb Friends' League" have acquired the lease of suitable premises near Victoria Station, where a hospital for animals will be erected.

The Duke and Duchess of Connaught were present last week at the annual festival of the Royal Normal College for the Blind, Upper Norwood. The Duke, who took the chair, commented on the excellent work of the institution, which provided for the training of the mind and body. Dr. Campbell, the principal, announced the receipt of purses containing £1,850.

An Accident Hospital has been opened at Denaby, which was erected by the efforts of over 4,000 workers engaged at the Denaby and Cadeby Main Collieries. They agreed to contribute a penny per man and a halfpenny per boy a week for the upkeep of the institution. The amount collected last year amounted to nearly £900. The site was presented by Mr. J. S. H. Fullerton. The accommodation consists of a ward for from six to ten beds, and a smaller ward for three beds. A verandah is provided on the south side of the wards. The cost of the building and furnishing has been £3,004. The architect is Mr. H. L. Smethurst.

Rather an interesting point as to allocating cost has arisen in the course of the provision of a small-pox hospital for Elginshire. Elginshire is inclined to depart from the general rule by which the proportion contributed is decided by valuation, and instead they propose to substitute a valuation based on population. One instance excepted, the proportion comes out very much the same. Not unnaturally, the district for whom the population basis acts unfavourably demurs to this method, on the plea that the heaviest burden falls on the poorest. An appeal to the Government Board leaves the matter for the locality to deal with. On the whole we are inclined to think that the valuation basis entails the least hardship. The matter is one which affects the whole county, and regarded in this light, it is only an adherence to the generally accepted principle that the richer shall help the poorer. It is also interesting to note that a stone and lime building can be secured in Elginshire for only £130 more than the cost of a composite wood building.

